# Semantic and right temporal variant of FTD: Next generation sequencing genetic analysis on a single-center cohort

**DOI:** 10.3389/fnagi.2022.1085406

**Published:** 2022-12-08

**Authors:** Giacomina Rossi, Erika Salvi, Elkadia Mehmeti, Martina Ricci, Cristina Villa, Sara Prioni, Fabio Moda, Giuseppe Di Fede, Pietro Tiraboschi, Veronica Redaelli, Cinzia Coppola, Giacomo Koch, Elisa Canu, Massimo Filippi, Federica Agosta, Giorgio Giaccone, Paola Caroppo

**Affiliations:** ^1^Neurology V and Neuropathology Unit, Fondazione IRCCS Istituto Neurologico Carlo Besta, Milan, Italy; ^2^Neuroalgology Unit, Fondazione IRCCS Istituto Neurologico Carlo Besta, Milan, Italy; ^3^Clinical Neuropsychology Unit, Fondazione IRCCS Istituto Neurologico Carlo Besta, Milan, Italy; ^4^Department of Advanced Medical and Surgical Sciences, University of Campania “L. Vanvitelli”, Naples, Italy; ^5^Non Invasive Brain Stimulation Unit/Department of Behavioral and Clinical Neurology, Santa Lucia Foundation IRCCS, Rome, Italy; ^6^Neuroimaging Research Unit, Division of Neuroscience, IRCCS San Raffaele Scientific Institute, Milan, Italy; ^7^Neurology Unit, IRCCS San Raffaele Scientific Institute, Milan, Italy; ^8^Vita-Salute San Raffaele University, Milan, Italy; ^9^Unit of Neurorehabilitation, IRCCS San Raffaele Scientific Institute, Milan, Italy; ^10^Neurophysiology Service, IRCCS San Raffaele Scientific Institute, Milan, Italy

**Keywords:** frontotemporal dementia, semantic variant, right temporal variant, next generation sequencing, genetic variant, pathogenic, mutation

## Abstract

Semantic and right temporal variant of frontotemporal dementia (svFTD and rtvFTD) are rare clinical phenotypes in which, in most cases, the underlying pathology is TDP-43 proteinopathy. They are usually sporadic disorders, but recent evidences suggest a higher frequency of genetic mutations for the right temporal versus the semantic variant. However, the genetic basis of these forms is not clear. In this study we performed a genetic screening of a single-center cohort of svFTD and rtvFTD patients, aiming at identifying the associated genetic variants. A panel of 73 dementia candidate genes has been analyzed by NGS target sequencing including both causal and risk/modifier genes in 23 patients (15 svFTD and 8 rtvFTD) and 73 healthy age-matched controls. We first performed a single variant analysis considering rare variants and then a gene-based aggregation analysis to evaluate the cumulative effects of multiple rare variants in a single gene. We found 12 variants in nearly 40% of patients (9/23), described as pathogenic or classified as VUS/likely pathogenic. The overall rate was higher in svFTD than in rtvFTD. Three mutations were located in *MAPT* gene and single mutations in the following genes: *SQSTM1, VCP, PSEN1, TBK1, OPTN, CHCHD10, PRKN, DCTN1*. Our study revealed the presence of variants in genes involved in pathways relevant for the pathology, especially autophagy and inflammation. We suggest that molecular analysis should be performed in all svFTD and rtvFTD patients, to better understand the genotype–phenotype correlation and the pathogenetic mechanisms that could drive the clinical phenotypes in FTD.

## Introduction

The term semantic variant of frontotemporal dementia (svFTD) usually refers to the form of FTD characterized by an early impairment of verbal semantic knowledge, that manifests as anomia and impaired single word comprehension, linked to the predominant atrophy of the left anterior temporal lobe and the diagnosis is based on current clinical criteria (Gorno-tempini et al., 2011). The right temporal variant of FTD (rtvFTD), a rarer clinical phenotype, originally considered a right variant of svFTD, has distinct clinical features and predominantly right hemisphere involvement. Patients with rtvFTD exhibit early behavioral symptoms, particularly empathy deficits and psychiatric disorders (Ulugut Erkoyun et al., 2020), but the diagnosis is challenging, and patients are often misdiagnosed as behavioral variant of FTD (bvFTD; Younes et al., 2022). A motor neuron involvement is rare in svFTD, but it has been found in up to 28% of rtvFTD cases in a recent neuropathological cohort (Ulugut et al., 2021).

While pathology can be highly heterogeneous in bvFTD, most of patients affected by svFTD and rtvFTD has TDP-43 neuronal accumulation, particularly the Type C pathology (Rohrer et al., 2010). In a minority of patients with rtvFTD a recent study reported also tauopathy as pathological substrate of the disease (Ulugut et al., 2021).

FTD has a prominent genetic basis, with up to 40% of cases presenting a family history (Mann and Snowden, 2017). To date three major genes have been described as linked to FTD: microtubule-associated protein tau (*MAPT*), progranulin (*GRN*) and Chromosome 9 open reading frame 72 (*C9ORF72*; Mann and Snowden, 2017). Furthermore, other causative genes have been disclosed, underlining rarer familial cases: Sequestosome 1 (*SQSTM1*), Valosin-Containing Protein (*VCP*), Charged Multivesicular Body Protein 2B (*CHMP2B*), TAR DNA Binding Protein, 43-KD (*TARDBP*), Fused in Sarcoma (*FUS*), Triggering Receptor Expressed on Myeloid cells 2 (*TREM2*), Tank-Binding Kinase 1 (*TBK1*) (see Fenoglio et al., 2018 for review). Left and right variants of FTD are usually sporadic, but recent evidence suggests a higher frequency of genetic mutations for the right versus the left variant (Ulugut Erkoyun et al., 2021). However, the genetic basis of these forms is not clear and genotype–phenotype correlations need to be elucidated, to allow accurate genetic counseling.

In this study, we used a next generation sequencing (NGS) approach to perform a genetic screening of a single-center cohort of patients with svFTD and rtvFTD, aiming at evaluating the genetic contribution in those disorders, by confirming candidate or discovering new genetic variants that could be associated with these clinical phenotypes.

## Materials and methods

### Cohort description

Twenty-three patients with clinical diagnosis of svFTD and rtvFTD (15 and 8 respectively) were retrospectively selected from FTD patients followed-up at Fondazione IRCCS Istituto Neurologico Carlo Besta from 2015 to 2020. One of them, carrying a *MAPT* mutation has been recently published (Villa et al., 2022). Clinical diagnosis of svFTD was made on the basis of current criteria (Gorno-Tempini et al., 2011). For the diagnosis of rtvFTD we followed the clinical and neuroimaging proposed framework (Ulugut Erkoyun et al., 2020). All patients underwent a complete clinical, neuropsychological and neuroimaging assessment. Analysis of markers of neurodegeneration (amyloid-β 1–42 – Aβ42, total tau and T181-phosphorylated-tau) was performed on cerebrospinal fluid (CSF) of 16 patients using LUMIPULSE G (Fujirebio, Malvern, PA, United States). Seventy-three age-matched healthy subjects from an in-house cohort were used as control group (healthy controls, HC). Written informed consent was obtained from all patient and healthy subjects.

### Next generation sequencing

DNA of patients was obtained, after informed consent, from peripheral blood lymphocytes. We designed a targeted enrichment panel to capture the coding and 25 bp flanking intron sequences of 73 dementia genes, including both causal genes and risk/modifier factors (Supplementary material S1). Nextera Flex for Enrichment system (Illumina, San Diego, CA, United States) coupled with gene-specific probes (Integrated DNA Technologies, Coralville, IA, United States) and the MiSeq instrument (Illumina, San Diego, CA, United States) were used for sequencing. A first quality control step was performed with FastQC software to identify base quality drops across cycles and adapter contamination, and to evaluate overall data quality. We performed both adapter trimming and quality trimming using Trimmomatic (version 0.36; Bolger et al., 2014). High-quality reads were mapped to the hg19 reference genome using bwa v. 0.7.17-r1188 (mem algorithm; Li and Durbin, 2010). Hence, we performed duplicated read marking, local realignment, and base quality score recalibration as suggested by GATK best practices (DePristo et al., 2011). We performed single-nucleotide variant (SNV) and insertion/deletion (INDEL) calling using the GATK module Haplotype Caller (version 4.1.9) over the target region. For the variant annotation we used the SnpEff software (Cingolani et al., 2012) that annotates and predicts the effects of genetic variants on genes and proteins. Variants that did not pass the variant filtering (total reads count≤20, alternative allele depth ≤ 10 and allele balance of 25%) were removed. Visual inspection of bam files was performed using IGV software (Thorvaldsdóttir et al., 2013).

### Single variant and gene-based aggregation analysis

In our cohort of 23 patients and 73 HC we first performed a single-variant analysis considering non-synonymous (missense, splicing, frameshift, STOP gain/loss) rare variants, that is variants with minor allele frequency (MAF) <0.01 in Genome Aggregation Database – Exomes – Non Finnish Europeans (GNOMEX_NFE). Franklin by Genoox^1^ and/or Varsome^2^ were used to report the classification according the criteria of the American College of Medical Genetics (ACMG).

Furthermore, we performed a gene-based aggregation analysis to evaluate the cumulative effects of multiple rare and low frequency (MAF <0.05) variants in a gene. All non-synonymous variants with MAF <0.05 in the studied cohort and reaching at least 80% of call rate were collapsed into single genes. Genes mapped by only one variant were not considered. To test whether there is an excess of variants in patients in comparison with HC, we applied both the combined burden and variance-component SKAT-O (Lee et al., 2012) and the simple burden method as implemented in EPACTS software (Efficient and Parallelizable Association Container Toolbox).^3^ Rho statistic indicates the direction of the effects, with rho = 1 referring to high percentage of causality in the same direction and rho = 0 to the simultaneous presence of causal and non-causal variants with opposing directions.

All the analyses were corrected for sex and age as covariates.

### Genotyping of *C9ORF72* exanucleotide repeat

The GGGGCC hexanucleotide repeat in the *C9ORF72* gene (DeJesus-Hernandez et al., 2011) was assessed by a repeat-primed PCR reaction using the AmplideX^®^ PCR/CE C9orf72 Kit (Asuragen, Austin, TX, United States), according to the manufacturer’s instructions. PCR products were analyzed on an ABI3130xl Genetic Analyzer.

## Results

Demographic and clinical data of overall cohort are reported in Table 1. Patients and HC did not show significant difference in term of sex (*p* = 0.11) and age (*p* = 0.18). 39% (9/23) of patients had a family history of dementia (53% of svFTD).

**Table 1:** Clinical and demographic features of study groups.

	Total	svFTD	rtvFTD	HC
N (M/F)	23 (13/10)	15(7/8)	8 (6/2)	73 (56/17)
Age at onset (mean±SD)	60.09 (±8.03)	60.27 (±8.69)	59.75 (±7.17)	–
Predominant symptom at onset		Loss of semantic knowledge	Memory impairment and behavioral alterations	–
MND	1/23	1/15	0	–
Family history of dementia	9/23	8/15	1/8	–
CSF (N)	16	11	5	
CSF Tau (pg/ml)	339.25 (±170.37)	351.36 (±193.27)	312.6 (±119.06)	
CSF p-tau (pg/ml)	38.32 (±19.85)	38.56 (±22.61)	37.65 (±11.61)	
CSF Abeta42 (pg/ml)	737.375 (±193.05)	783.45 (±177.46)	636 (±205.77)	

FTD = Frontotemporal dementia; sv = semantic variant; rtv = right temporal variant; HC = healthy controls; SD = standard deviation; N = number; M = male; F = female; MND = Motor neuron disease; CSF = cerebrospinal fluid. Patients and HC did not show significant difference in term of sex (*p* = 0.11) and age (*p* = 0.18).

Semantic impairment with naming deficits was the first symptom of svFTD patients, while rtvFTD patients showed early changes in their personality and behaviour (among them, eating behavior changes, loss of empathy, compulsive behaviors), memory loss, prosopagnosia and topographical disorientation. According with clinical diagnosis, brain magnetic resonance imaging and fluorodeoxyglucose-positron emission tomography showed predominant atrophy and hypometabolism in left anterior temporal lobe in svFTD and a predominant right temporal involvement in all rtvFTD. CSF biomarkers were in the normal range in 13/16 patients, 3 patients had isolated reduction of Abeta42. Total tau was elevated in 2 patients and one of them had also elevated p-tau, but normal Abeta42.

### Single variant analysis

By single variant analysis, we found 12 variants described as pathogenic in the scientific literature (reported in Human Gene Mutation Database, HGMD) or classified as variant of unknown significance (VUS)/likely pathogenic in 9 patients (39% of patients) and none in control subjects (Table 2).

**Table 2:** Variants present in patients.

Patient	FTD	Gene	Codon	Reference	Associated disease (HGMD)	ACMG	Notes on pathogenicity	AF_GNOMEX_NFE
	(rtv/sv)							
P01	rtv	*MAPT*	N621N (N286N)*	NM_001123066.3:c.1863C > T	nr	Likely benign	Splicing enhancer alteration	2.38E-05
		*PSEN1*	M93V	NM_000021.3:c.277A > G	nr	Likely pathogenic	Within a mutational hot spot	8.95E-06
P05	sv	*MAPT*	P636L (P301L)*	NM_001123066.3:c.1907C > T	FTD	Pathogenic	Several reports on genetics, function, neuropathology, animal models	1.32E-05
P06	sv	*OPTN*	Q314L	NM_021980.4:c.941A > T	ALS	VUS-LP	15 pathogenicity scores^$^	2.78E-04
		*DCTN1*	R795H	NM_004082.4:c.2384G > A	CMT	VUS-LP	18 pathogenicity scores^$^	4.48E-05
P10	rtv	*CHCHD10*	P80L	NM_213720.1:c.239C > T	ALS	VUS	9 pathogenicity scores^$^	2.31E-04
P11	sv	*PRKN*	T240M	NM_004562.2:c.719C > T	PD	Pathogenic	Functional studies	2.51E-04
P12	sv	*MAPT*	Q671H (Q336H)*	NM_001123066.3:c.2013G > T	FTD	VUS-LP	Reports on genetics, function, neuropathology	0
P20	sv	*SQSTM1*	E280/del	NM_003900.4:c.838_840delGAG	FTD	VUS-LP	Susceptibility factor	1.79E-05
P21	sv	*VCP*	G376E	NM_007126.3:c.1127G > A	nr	VUS-LP	New, absent in population databases, 19 pathogenicity scores^$^	0
		*TBK1*	I207T	NM_013254.3:c.620 T > C	ALS	VUS	10 pathogenicity scores^$^	1.08E-04
P23	sv	*SQSTM1*	P387L	NM_003900.4:c.1160C > T	FTD, ALS, PBD	VUS-LP	Reports on genetics	5.97E-04

FTD = Frontotemporal dementia; rtv = right temporal variant; sv = semantic variant;

* NM_005910; HGMD = Human Gene Mutation Database; nr = not reported; ALS = Amyotrophic lateral sclerosis; CMT = - Charcot–Marie-Tooth; PD = Parkinson’s disease; PDB =. Paget disease of bone; ACMG = American College of Medical Genetics; VUS = variant of unknown significance; VUS-LP = VUS near likely pathogenic.

$ https://varsome.com; AF_GNOMEX_NFE = Allele frequency from Genome Aggregation Database - Exome - Non Finnish Europeans.

We found three mutations in the microtubule-associated protein tau (*MAPT*) gene, which was the first causative gene to be linked to FTD: P301L, Q336H (Villa et al., 2022) and N286N. In addition, we discovered single mutations in the following genes: *SQSTM1*, *VCP*, presenilin 1 (*PSEN1*), *TBK1*, optineurin (OPTN), coiled-coil-helix-coiled-coil-helix domain containing protein 10 (*CHCHD10*), parkin (*PRKN*) and dynactin 1 (*DCTN1*). Three patients carried two mutations each: P01, N286N *MAPT* and M93V *PSEN1*; P06, Q314L *OPTN* and R795H *DCTN1*; P21, G376E *VCP* and I207T *TBK1*. All the variants are singletons (present only in 1 patient) and not present in control subjects. Clinical characteristics of the 9 patients carrying the variants are reported in Table 3.

**Table 3:** Clinical and demographic characteristics of the 9 patients carrying the variants.

	*P01*	*P05*	*P06*	*P10*	*P11*	*P12*	*P20*	*P21*	*P23*
*Clinical variant*	rtv	sv	sv	rtv	sv	sv	sv	sv	sv
*Gender*	M	M	F	M	M	F	F	M	F
*Family history*	pos	pos	neg	neg	pos	pos	neg	pos	pos
*Age at onset*	50	47	59	67	65	37	63	61	58
*Disease duration*	5	12	na	5	8	7	6	6	2
*Gene variant*	*MAPT* N286N	*MAPT* P301L	*OPTN* Q314L	*CHCHD10* P80L	*PRKN* T240M	*MAPT* Q336H	*SQSTM1* E280/del	*VCP* G376E	*SQSTM1 P387L*
	*PSEN1* M93V		*DCTN1* R795H					*TBK1* I207T	
*CSF analysis/amyloid-PET*	PET-amyloid neg	Normal CSF	Normal CSF	Normal CSF	Normal CSF	Reduced Aβ42,	na	Normal Aβ42, elevated tau and p-tau	na
						normal tau and p-tau			
*MMSE (/30)*	18*	4*	25	27*	na	9*	26	12*	na
*MoCA (/30)*	na	na	na	na	na	na	na	na	22*
*FAB (/18)*	13*	na	na	12*	na	9*	15	4*	16
*FBI (/72)*	33	na	na	14	na	21	12	8	na

rtv = right temporal variant; sv = semantic variant; na = not available; CSF = cerebrospinal fluid; PET = positron emission tomography; MMSE = Mini-Mental State Examination; MoCA = Montreal Cognitive Assessment; FAB = Frontal Assessment Battery; FBI = Frontal Behavioral Inventory.

* under cut-off; pos = positive; neg = negative. For *MAPT*, reference NM_005910 is used.

### Gene-based aggregation analysis

None of the genes reached the Bonferroni corrected threshold (for the total number of genes considered, *p* = 0.001). However, the SKAT-O test highlighted 4 genes with suggestive value of *p* <0.05, enriched for rare variants: *CD33*, *PSEN1*, *OPTN* and *ABCA1* (Table 4).

**Table 4:** Genes enriched by rare/low frequency variants after gene-based analysis with a SKAT-O *p* value <0.05.

			SKAT-O		BURDEN	
GENE	N tested variants	N Singleton variants	Rho	Value of *p*	Value of *p*	OR
*CD33*	4	2	0.1	3.28E-03	2.71E-02	4.57
*PSEN1*	3	2	0.1	2.99–02	3.60E-02	5.91
*OPTN*	2	1	1	4.46E-02	3.95E-02	7.89
*ABCA1*	9	6	0	4.83E-02	2.61E-01	0.56

SKAT-O, Optimized sequence kernel association test; OR, Odds Ratio. The results are ordered by SKAT-O p-value. Rho statistic indicates the direction of the effects, with rho = 1 referring to high percentage of causality in the same direction and rho = 0 to the simultaneous presence of causal and non-causal variants with opposing directions.

*OPTN* with a rho value equal to 1 showed a unidirectional risk association, reflected by the fact that the two rare variants are exclusively present or more frequent in cases than in HC: M98K was present in 6.5% of patients and 2.1% of HC, whereas Q314L was carried by one patient (Table 5). The simple burden test showed *OPTN* as conferring a risk 7.89-fold higher in patients compared to HC (*p* = 0.0395, OR = 7.89).

**Table 5:** Variants present in the enriched genes (gene-based aggregation analysis).

Gene	codon	Reference	Associated disease (HGMD)	ACMG	AF_GNOMEX_NFE	Cohort MAF	% pts	% HC	N pts (*n* = 22)	N HC (*n* = 73)
*CD33*	S305P	NM_001772.3:c.913 T > C	nr	benign	2.15E-02	2.60%	8.70%	0.70%	4	1
*CD33*	G156fs	NM_001772.3:c.466_469delGGCC	AD risk	benign	2.43E-02	2.60%	2.20%	2.70%	1	4
*CD33*	F243L	NM_001772.3:c.727 T > C	nr	benign	3.58E-04	0.50%	2.20%	0.00%	1	0
*CD33*	V267I	NM_001772.3: c.799G > A	nr	benign	4.39E-04	0.50%	2.20%	0.00%	1	0
*PSEN1*	E318G	NM_000021.3:c.953A > G	AD risk	benign	1.86E-02	1.60%	4.30%	0.70%	2	1
*PSEN1*	M93V	NM_000021.3:c.277A > G	nr	Likely pathogenic	8.95E-06	0.50%	2.20%	0.00%	1	0
*PSEN1*	D333V	NM_000021.3:c.998A > T	nr	Likely pathogenic*	0	0.50%	0.00%	0.70%	0	1
*OPTN*	M98K	NM_001008211.1:c.293 T > A	glaucoma risk	Likely benign	2.80E-02	3.10%	6.50%	2.10%	3	3
*OPTN*	Q314L	NM_001008211.1:c.941A > T	ALS	VUS-LP	2.78E-04	0.50%	2.20%	0.00%	1	0
*ABCA1*	Q2196H	NM_005502.3:c.6588G > C	HDL deficiency	VUS-LP	2.25E-04	0.50%	2.20%	0.00%	1	0
*ABCA1*	R909Q	NM_005502.3:c.2726G > A	nr	VUS	2.69E-05	0.50%	0.00%	0.70%	0	1
*ABCA1*	T774P	NM_005502.3:c.2320A > C	Increased cholesterol	Likely benign	3.15E-03	0.50%	0.00%	0.70%	0	1
*ABCA1*	M674L	NM_005502.3:c.2020A > C	nr	VUS	0	0.50%	2.20%	0.00%	1	0
*ABCA1*	V285M	NM_005502.3:c.853G > A	nr	VUS	1.79E-05	0.50%	2.20%	0.00%	1	0
*ABCA1*	.	NM_005502.3:c.814-7A > G	nr	VUS	.	0.50%	2.20%	0.00%	1	0
*ABCA1*	K776N	NM_005502.3:c.2328G > C	Risk ischemic heart disease	Benign	3.38E-03	1.00%	0.00%	1.40%	0	2
*ABCA1*	E1172D	NM_005502.3:c.3516G > C	Coronary heart disease	Benign	2.90E-02	4.20%	0.00%	5.50%	0	7
*ABCA1*	V771M	NM_005502.3:c.2311G > A	Altered cholesterol levels	Benign	3.32E-02	4.70%	0.00%	6.20%	0	8

HGMD = Human Gene Mutation Database; nr = not reported; ALS = Amyotrophic lateral sclerosis; AD = Alzheimer’s disease; HDL = high-density lipoprotein; ACMG = American College of Medical Genetics; VUS = variant of unknown significance; VUS-LP = VUS near likely pathogenic; AF_GNOMEX_NFE = Allele frequency from Genome Aggregation Database - Exome - Non Finnish Europeans. MAF = Minor allele frequency;

* for cardiovascular disease; N = number; pts = patients; HC = Healthy controls.

A rho value equal to 0.1, for *CD33* and *PSEN1*, indicated a simultaneous presence of causal and non-causal variants with opposing directions. *CD33* is enriched of 4 rare/low frequency variants in 7 patients and 5 HC: the frameshift G156fs was shared by patients and HC with a similar frequency, S305P was present in 4 of 23 patients (8.7%) and one HC (0.7%), F243L and V267I were exclusively carried by one patient (Table 5). In *PSEN1*, E318G was found in 4.3% of patients and 0.7% of controls, the M93V was exclusively carried by one patient whereas D333V was present in one control.

As for *ABCA1* (Table 5), a rho value of 0 indicates the simultaneous presence of causal and non-causal variants with opposing directions. In fact, out of 9 variants, 4 were present in patients and 5 in a high number of HC, showing opposite directions in terms of risk association.

### Genotyping of *C9ORF72* exanucleotide repeat

None of the patients carried an expanded allele (>30 repeats).

## Discussion

In this study we found several pathogenic or VUS/likely pathogenic variants, in nearly 40% of patients.

P301L, carried by P05, is one of the most known and studied *MAPT* mutations, whose pathogenicity has been demonstrated in several genetic, functional and neuropathological studies, as well as in animal models (Hutton et al., 1998; Barghorn et al., 2000; Lewis et al., 2000; Rossi et al., 2008). Different FTD clinical phenotypes have been associated with the mutation, including svFTD (Ishizuka et al., 2011). Q336H *MAPT* mutation, carried by patient P12, was recently published as associated with svFTD (Villa et al., 2022). This mutation had been previously functionally and neuropathologically characterized as pathogenic (Tacik et al., 2015). Patient P01 carried the N286N *MAPT* and M93V *PSEN1* variants. As for the N286N *MAPT* variant, only once cited without additional details (Rohrer et al., 2009), the classification as likely benign does not take into account the pathogenetic mechanism typical of some silent *MAPT* mutations localized in exon 10, that is the alteration of an exon 10 splicing enhancer or silencer (D’Souza and Schellenberg, 2002; Qian and Liu, 2014). In fact, an *in silico* functional analysis demonstrated the effect on exon 10 splicing of this variant (Tubeuf et al., 2020). The M93V variant in *PSEN1*, so far never described, is very interesting as localized in a mutational hot spot in a critical region for the protein function, the transmembrane domain 1: in fact, the adjacent mutations C92S (Zhang et al., 2000; Tedde et al., 2003) and V94M (Arango et al., 2001; Somavarapu and Kepp, 2016) are associated with Alzheimer’s disease (AD). In addition, several other pathogenic AD mutations have been described in the same protein region. It is well known that *PSEN1* mutations give rise to Alzheimer’s disease by affecting the beta amyloid levels; however, a few mutations have been reported as associated with a FTD phenotype, even pathologically confirmed as a tauopathy (Dermaut et al., 2004), although how loss of functional presenilin 1 could predispose to tauopathy has not been clarified. Our patient had a clinical phenotype of rtvFTD with early memory deficits and behavioural disturbances including lack of empathy, hyperphagia and loss of inhibition, the clinical phenotype suggesting the prevalence of FTD neurodegeneration pathway, while the AD pathway may cooperate in the pathology. Moreover, amyloid-PET was negative in our patient, making AD diagnosis unlikely.

*MAPT* mutations have been previously identified in 4 rtvFTD cases (Ulugut Erkoyun et al., 2021). However, in our cohort, both svFTD (P05 and P12) and rtvFTD (P01) patients carried *MAPT* mutations, not confirming a prevalent association of *MAPT* mutations with rtvFTD.

Patient P06 had Q314L *OPTN* and R795H *DCTN1* variants. *OPTN*, earlier reported as causative of primary open-angle glaucoma, was afterwards linked to ALS (Maruyama et al., 2010), or to FTD (Dominguez et al., 2021), with dominant or recessive transmission. Heterozygous Q314L in *OPTN* was described in some sporadic ALS cases (Del Bo et al., 2011; Pensato et al., 2020). *DCTN1*, firstly linked to ALS and Perry syndrome, was then associated with progressive supranuclear palsy and FTD phenotypes (Caroppo et al., 2014). R795H variant was only described in a case of Charcot–Marie-Tooth. These variants are classified as VUS-near likely pathogenic and may contribute to the FTD pathology in our patient, although with very different mechanisms, as *OPTN* is involved in autophagy whereas *DCTN1*, binding to microtubules and to dynein, has a role in retrograde axonal transport (LaMonte et al., 2002). Patient P21 carried the G376E *VCP* and I207T *TBK1* variants. Mutations in *VCP* were identified as the cause of inclusion body myopathy with Paget disease of bone and frontotemporal dementia (IBMPF); (Watts et al., 2004). Afterwards, *VCP* mutations were also found in cases of pure FTD (Saracino et al., 2018). The G376E variant has never been described and is not present in population databases, constituting a possible new mutation. In fact, it is adjacent to the ATPase domain 1, where other mutations have been described (Mol et al., 2021), and it is classified as VUS-near likely pathogenic, having at least 19 pathogenicity scores. I207T in *TBK1* was described in a patient affected by sporadic ALS (Tohnai et al., 2018). The mutation is within the serine/threonine kinase domain and has 10 pathogenicity scores. *TBK1* is now recognized also as a FTD gene (van Mossevelde et al., 2018). Since *VCP* has several functions, including the maintenance of lysosomal homeostasis (Arhzaouy et al., 2019), it may cooperate with *TBK1*, whose principal role is in the autophagy. The coexistence of variants in genes involved in the same pathway reinforces their pathogenic role and may explain the pathology. Both P06 and P21 patients were svFTD, without any sign of ALS.

Patients P20 and P23, both svFTD, carried a *SQSTM1* mutation. *SQSTM1* mutations were firstly identified as causative of Paget Disease of Bone (PDB; Laurin et al., 2002), and afterwards this gene was disclosed to be also causative of ALS (Fecto et al., 2011) and FTD (Le Ber et al., 2013). The P387L mutation, carried by patient P23, was previously described in patients affected by PDB (Johnson-Pais et al., 2003), and also in familiar cases of FTD, where a segregation with the disease was demonstrated (Le Ber et al., 2013). The amino acid deletion E280, carried by patient P20, was described as a susceptibility factor for FTD (Van Der Zee et al., 2014).

Patient P10, rtvFTD, had the P80L *CHCHD10* mutation, previously reported in patients affected by familial or sporadic ALS (Ronchi et al., 2015; Zhang et al., 2015). *CHCHD10* codes for a mitochondrial protein associated not only with ALS but also with FTD (Chaussenot et al., 2014).

While homozygous or heterozygous compound mutations in *PRKN* lead to early-onset Parkinson’s disease (PD; Lücking et al., 2000), the role of heterozygous mutations is still controversial, ranging from benign condition to susceptibility factor (Marder et al., 2010; Moura et al., 2013). *PRKN* T240M mutation, carried by the svFTD patient P11, is predicted to eliminate a phosphorylation site for casein kinase II and occurs in the same codon as other mutations (T240R and T240K), indicating that this is an important functional residue. Furthermore, functional studies in neuronal cell cultures demonstrated that this mutation impairs glutamatergic signaling (Zhu et al., 2018). Biallelic mutations in *PKRN* can lead to FTD (Zimmermann et al., 2018), whereas we suppose that heterozygous mutations may only constitute a susceptibility factor.

In summary, as for single variant findings, we found 12 mutations in 9 patients out of 23, evidencing a strong genetic component in FTD phenotypes usually regarded as sporadic. We also showed that the mutations appeared to be more associated with svFTD (7/15, 47%) than with rtvFTD (2/8, 25%) at variance with what suggested by others (Ulugut Erkoyun et al., 2021). Our data show that a gene strongly involved in our cohort of patients is *MAPT*, one of the three major genes causative of the FTD spectrum. Along with *DCTN1*, the involvement of this gene clearly demonstrates the relevance of the correct microtubule dynamics and transport in the nervous system. The mutations found in our patients outline cell pathways whose alterations are common to different neurodegenerative diseases such as FTD, ALS and Parkinson’s disease (PD). Lysosomal and autophagic functions are sustained by *VCP*, which plays a role in lysosome homeostasis, and by *SQSTM1*, *TBK1* and *OPTN*, which interact in the autophagy (Abramzon et al., 2020; Fleming et al., 2022), all mutated in ALS and FTD. In addition, mitophagy, a specialized form of autophagy concerning mitocondria, is carried out by the interaction of *TBK1*, *PRKN* and *OPTN* (Harding et al., 2021). Mitochondrial homeostasis is the function of *CHCHD10*, linked to FTD, ALS and PD (Jiang et al., 2022).

We also performed a gene-level association analysis (burden analysis) in order to disclose genes which may contain a significantly higher number of variants in patients than in controls, suggesting the gene to be relevant to the pathology. We are aware of the small size of our cohorts, partly justified by the rarity of the disease, and in fact we did not achieved the Bonferroni significant threshold, but only suggestive *p* values highlighting 4 genes: *CD33, PSEN1* and *ABCA1*, showing a simultaneous presence of causal and non-causal variants with opposing directions, and *OPTN,* showing unidirectional risk association with rare variants exclusively present or more frequent in patients than in HC.

CD33 is a transmembrane protein and a sialic acid-binding immunoglobulin-like lectin that regulates innate immunity. In the CNS, it is expressed by the microglia. CD33 has an immunoreceptor tyrosine-based inhibitory motif (ITIM) and one ITIM-like domain that facilitates an inhibitory signal. When CD33 is activated by sialic-acid-containing glycoproteins and glycolipids, it results in inhibition of microglia phagocytosis. Genome-wide association studies (GWAS) identified several genetic loci associated with increased susceptibility to late onset Alzheimer’s disease, including CD33 (Naj et al., 2011). Some polymorphisms increase CD33 expression, giving rise to reduced microglial phagocytosis and amyloid clearance. Although most polymorphisms were related to AD dementia, others were described as associated with a FTD phenotype (Rendina et al., 2020). In our cohorts we found 4 CD33 rare or low frequency variants. In particular, S305P was found in 9.1% of patients and 0.7% of control subjects, while F243L and V267I were only found in one patient each and no controls. G156fs was found approximately with the same frequency in patients and controls. All these variants are classified as benign. Besides the involvement in amyloid clearance, a pathological feature of AD, CD33 may be regarded as a gene more generally involved in immune and inflammatory pathways, which are also altered in FTD (Ferrari et al., 2014, 2015).

We have already discussed the contribution of a *PSEN1* likely pathogenic variant, M93V, to FTD phenotype (see above); in addition, the gene-based analysis revealed the presence of the E318G variant, previously described as a risk factor for AD but afterwards producing conflicting results (Dermaut et al., 1999). The D333V variant, present in a control subject, was never reported before and it is classified as likely pathogenic for cardiovascular disease (Li et al., 2006). As for *OPTN*, we have already described the Q341L variant (see above), while the M98K is reported in databases as likely benign.

*ABCA1* is an ATP-binding cassette transporter that controls whole brain cholesterol homeostasis. Polymorphisms in *ABCA1* have been demonstrated to influence susceptibility to dementia, in particular to AD (Lupton et al., 2014; Chen et al., 2016). In our cohorts we found 9 *ABCA1* rare or low frequency variants. Four of them were found in patients and the others in controls, showing opposite directions in terms of risk association, that is not giving a true causal significance to this gene as regards to FTD phenotypes. Literature data about *ABCA1* concern primarily cardiovascular disease and variants classified as benign due to their relatively high frequency in the population are often associated with a certain level of risk for these diseases. As for dementias, in experimental models, *ABCA1* activities appear to influence neuroinflammation and neurodegeneration (Karasinska et al., 2013), thus possibly affecting different forms of dementia, including FTD.

In conclusion, our study revealed the presence of variants in genes involved in pathways relevant for the pathology, especially autophagy and inflammation, common to different neurodegenerative diseases such as FTD, ALS, AD and PD. The findings support the role of these pathways in semantic phenotypes of FTD, as recently suggested (Pascual et al., 2021). Molecular analysis of dementia-related genes should be performed in all patients with svFTD and rtvFTD, to better understand the genotype–phenotype correlations and to study the pathogenetic mechanisms that could drive the clinical phenotypes in FTD.

## Data availability statement

The original contributions presented in the study are included in the article/Supplementary material, further inquiries can be directed to the corresponding author.

## Ethics statement

The studies involving human subjects were reviewed and approved by Ethics Committee of Fondazione IRCCS Istituto Neurologico Carlo Besta (Prot. n.39–05/04/2017). All the subjects provided their written informed consent to participate in this study.

## Author contributions

GR and PC: conceptualization. ES, EM, and MR: formal analysis. CV, SP, FM, GF, PT, VR, CC, GK, EC, MF, and FA: investigation. FA and GG: funding acquisition. GR, ES, and EM: methodology. GR, PC, and ES: writing – original draft. GR, PC, and FA: writing – review and editing. All authors contributed to the article and approved the submitted version.

## Funding

This research was funded by the European Research Council (StG-2016_714388_NeuroTRACK) and the Italian Ministry of Health, Ricerca Corrente RRC.

## Conflict of interest

EC has received research supports from the Italian Ministry of Health. MF is Editor-in-Chief of the *Journal of Neurology*; received compensation for consulting services and/or speaking activities from Bayer, Biogen Idec, Merck-Serono, Novartis, Roche, Sanofi Genzyme, Takeda, and Teva Pharmaceutical Industries; and receives research support from Biogen Idec, Merck-Serono, Novartis, Roche, Teva Pharmaceutical Industries, Italian Ministry of Health, Fondazione Italiana Sclerosi Multipla, and ARiSLA (Fondazione Italiana di Ricerca per la SLA). FA is Section Editor of *NeuroImage: Clinical*; has received speaker honoraria from Biogen Idec, Roche and Zambon; and receives or has received research supports from the Italian Ministry of Health, AriSLA (Fondazione Italiana di Ricerca per la SLA), the European Research Council and the Foundation Research on Alzheimer Disease.

The remaining authors declare that the research was conducted in the absence of any commercial or financial relationships that could be construed as a potential conflict of interest.

## Publisher’s note

All claims expressed in this article are solely those of the authors and do not necessarily represent those of their affiliated organizations, or those of the publisher, the editors and the reviewers. Any product that may be evaluated in this article, or claim that may be made by its manufacturer, is not guaranteed or endorsed by the publisher.

## Supplementary material

The Supplementary material for this article can be found online at: https://www.frontiersin.org/articles/10.3389/fnagi.2022.1085406/full#supplementary-material

## Footnotes

^1^ https://franklin.genoox.com/clinical-db/home

^2^ https://varsome.com

^3^ http://genome.sph.umich.edu/wiki/EPACTS
