# Supplementary material for: Semantic and right temporal variant of FTD: Next generation sequencing genetic analysis on a single-center cohort
Source: Front Aging Neurosci. 2022 Dec 8;14:1085406. doi: 10.3389/fnagi.2022.1085406 (PMC9773257; doi:10.3389/fnagi.2022.1085406)
Supplement: Supplementary file 1 [file Table_1.DOCX]

**Supplementary File S1**: list of genes analyzed in the NGS panel

ABCA1

ABCA7

ADAM10

AKAP9

APOE

APP

BCL7C

BIN1

CALHM1

CCL2

CCNF

CD2AP

CD33

CHCHD10

CHMP2B

CLU

CSF1R

CST3

CTSF

CXCR4

DCTN1

EIF4G1

EPHA1

FLNC

FUS

GABRB3

GBA

GIGYF2

GRN

GSN

hnRNPA1

hnRNPA2B1

ITM2B

LRRK2

MAPT

NCSTN

NOS3

NOTCH3

NPC1

NPC2

OPTN

PFN1

PICALM

PINK1

PLD3

PRKAR1B

PRKN

PRNP

PSEN1

PSEN2

SCARB2

SERPINI1

SIGMAR1

SNCA

SNCB

SOD1

SORL1

SORT1

SQSTM1

STH

STX1B

TARDBP

TBK1

TIA1

TMEM106B

TREM2

TSC1

TTR

TUBA4A

TYROBP

UBQLN2

UNC5C

VCP
